# Wave dispersion in moderate channel turbulence

**DOI:** 10.1038/s41598-023-32978-7

**Published:** 2023-04-26

**Authors:** Chiara Pilloton, Claudio Lugni, Giorgio Graziani, Francesco Fedele

**Affiliations:** 1 CNR-INM, Institute of Marine Engineering, Via di Vallerano 139, 00128 Roma, Italy; 2 School of Civil and Environmental Engineering, Georgia Institute of Technology, Atlanta, USA; 3 Department of Mechanical and Aerospace Engineering, Sapienza Universitá di Roma, Roma, Italy; 4 NTNU-AMOS, Center for Autonomous Marine Operation Systems, Trondheim, Norway; 5 Institute of Marine Hydrodynamics, Harbin Engineering University, Harbin, China

**Keywords:** Fluid dynamics, Ocean sciences, Statistics

## Abstract

We study channel turbulence by interpreting its vorticity as a random sea of ocean wave packet analogues. In particular, we investigate the ocean-like properties of vortical packets applying stochastic methods developed for oceanic fields. Taylor’s hypothesis of frozen eddies does not hold when turbulence is not weak, and vortical packets change shape as they are advected by the mean flow, altering their own speed. This is the physical manifestation of a hidden wave dispersion of turbulence. Our analysis at the bulk Reynolds number Re_*b*_ = 5600 suggests that turbulent fluctuations behave dispersively as gravity-capillary waves, with capillarity being dominant near the wall region.

## Introduction

Unlike laminar flows where particles have smooth and highly ordered paths, turbulent channel flows are characterized by velocity fluctuations and a highly disordered motion. Turbulent flow is generally dominated by a chaotic motion of fluid particles that are advected in an irregular flow varying in space and time. This observation led Taylor^1^ to describe a turbulent flow as filled by frozen vortices that are advected downstream by the mean flow. This is Taylor’s hypothesis, which essentially holds in the weak turbulence regime, when flow fluctuations around the mean are small compared to the large-scale flow. Then, eddies can be considered as passive scalars transported at the mean flow velocity, or Taylor speed. However, when the turbulent flow is not weak, eddies undergo a distortion of their shapes as they are advected by the mean stream. Such shape-changing dynamics can speed up or slowdown the vortices, while being advected at a different speed than the mean flow velocity^2–6^. Thus, the speed of coherent turbulent structures includes not only a dynamical velocity, which accounts primarily for their inertia, but also a geometric component. This can be interpreted as a self-propulsion velocity^2^ induced by the shape-changing deformations of the flow structures, similar to that of a low Reynolds number swimmer^7^. In this case, the dynamical velocity is null since inertia is neglected and the swimmer’s velocity is uniquely determined by the geometry of the sequence of its body’s shapes. In a fixed laboratory frame, we observe the swimmer drifting as its body’s shape varies in time. In a frame moving with the swimmer we just observe his body’s shape variations. In wave mechanics, the recently noticed slowdown effect of crests of dominant oceanic wave groups^8^ can be explained in terms of geometric phase velocities^9^. In particular, in deep waters the largest wave of a group tends to slow down as it reaches the maximum height at focus and then speed up^10^. On the contrary, in capillary wave groups the largest crest tends to speed up before focus and then slow down. In both cases, the crest of the wave group changes its shape while slowing down or speeding up, as the kinematic manifestation of the natural dispersion of surface gravity waves^10^. Such a behaviour is analogous to that of turbulent vortices that change their shape as they are advected by the flow.

In this work we study channel flow turbulence by interpreting its vorticity as a random sea of ocean wave groups analogues. We then investigate the space-time evolution of its vortical packets and their wave-like properties supported by direct numerical simulations (DNS, hereafter). In this regard, it is well established that Self-Sustaining Processes (SSP)^11–13^ capture the essence of turbulent structures in channel flows: streamwise vortical rolls remodulate the streamwise velocity in the form of streaks by redistributing the momentum in the crossflow planes. The streaks are unstable to spanwise disturbances leading to a streamwise ondulation, which regenerates the streamwise rolls via a nonlinear self-interaction, and the process repeats sustaining itself^11,12^. Thus, in this work we will investigate the dispersive properties of both spanwise and streamwise vorticity fields. To do so, we use stochastic methods developed for random oceanic fields, in particular the theory of quasi-determinism (QD) for ocean waves^14–17^. We aim at verifying the hypothesis that Navier–Stokes turbulence hides an ocean-like dispersion behaviour that governs the shape-changing dynamics of vortices, in analogy with the evolution of oceanic wave groups^9,10^.

The paper is structured as follows. We first briefly overview the theoretical results on the wave dispersion of axisymmetric turbulent flows^18^. Then, we introduce the case study of DNS turbulent channel flows simulations, and then overview Taylor’s hypothesis^1^ in relation to our work. We then provide evidence that when turbulence is not weak, deviations from Taylor’s hypothesis relate to a hidden wave-like dispersion of turbulence in analogy with surface gravity waves. In doing so, we first review the dispersive properties of surface gravity wave packets of an ocean field. Then, we investigate the kinematics of the analogous wave packets of vorticity in turbulent channel flows. We then conclude by discussing our results and findings.

## Wave dispersion in axisymmetric Navier–Stokes flows

**Figure 1:**
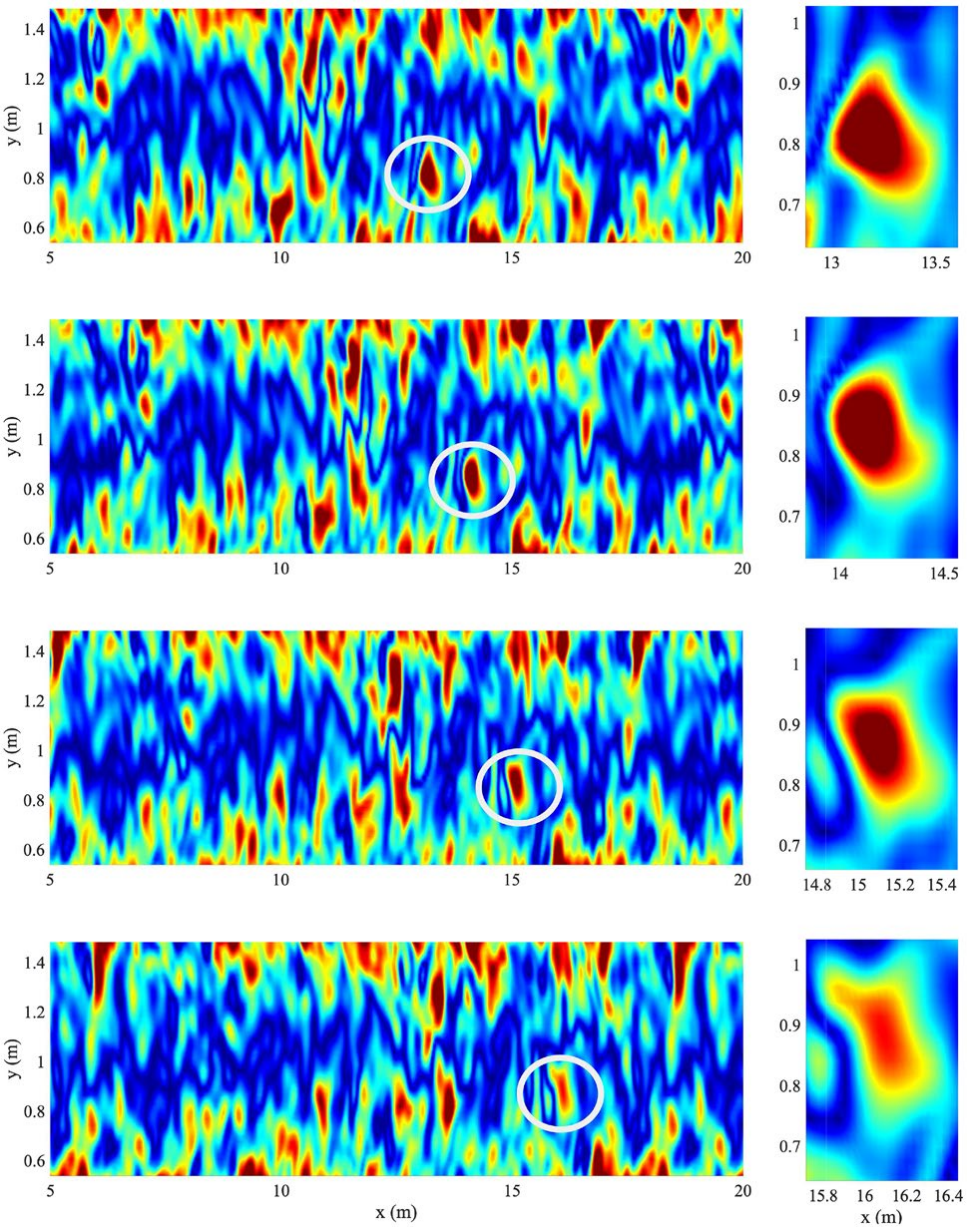
Left: snapshots of the average spanwise vorticity field $\overline{\omega }_z(x,y,t)$ and tracking of the encircled eddy. Right: details of the shape of the encircled eddy. Time increases from top to bottom, related animation Movie S1.

Fedele and Dutykh^18,19^ investigated the dynamics of non-rotating axisymmetric pipe flows in terms of nonlinear soliton bearing equations. They showed that at high Reynolds numbers, the dynamics of perturbations to the laminar flow obey a coupled system of nonlinear Camassa–Holm (CH) equations^20^. These support inviscid and smooth localized travelling waves in the form of localized toroidal vortices that concentrate near the pipe boundaries (wall vortexons) or wrap around the pipe axis (centre vortexons) in agreement with the analytical soliton solutions derived by Fedele^21^ for small and long-wave disturbances and the nonlinear neutral structures derived by Walton^22^. Such an azimuthal vorticity is the analogue of the spanwise vorticity in channel flows considered in this work (see Fig. 1). Azimuthal vorticity in the pipe splits into a centre vortexon radiating vorticity in the form of wall vortexons. These can under go further splitting before viscosity dissipates them, leading to a slug of centre vortexons. The splitting process originates from a radial flux of azimuthal vorticity from the wall to the pipe axis^23^.

Drawing on Fedele and Dutykh^18,19,21^ consider the axisymmetric perturbation to a laminar pipe fluid flowing in the streamwise direction *x*, with *r* the radial direction. The associated streamfunction is $\psi \sim A(x,t)\phi (r)$, where $\phi (r)$ is the least stable eigenmode of the linearized axisymmetric Navier–Stokes equations around the laminar flow solution^21^ and the amplitude *A*(*x*, *t*) satisfies a CH type equation^18^

1 $$\begin{aligned} \partial _t A + u_m\,\partial _x A - \alpha \, \partial _{xxt} A - \gamma \, \partial _{xxx} A -\beta \partial A_{xxxxx} + F\, A\,\partial _x A +H\, A\,\partial _{xxx} A= 0, \end{aligned}$$

where $u_m$ is a mean advection velocity, $(\alpha ,\gamma ,\beta )$ are free parameters, (*F*, *H*) are the coefficients of the nonlinear terms and *r* is the radial direction. Here, we added a fifth-order dispersion term ruled by $\beta$ to model dispersive effects beyond axisymmetry. Linearizing around the constant steady state solution $A=A_0$, the associated phase speed of a Fourier wave perturbation $e^{i(k x - \omega t)}$ is

2 $$\begin{aligned} C(k) = \frac{\omega (k)}{k} = \frac{u_0 + \alpha C_0 k^2 - \beta k^4}{1 + \alpha k^2}, \end{aligned}$$

where $u_0=u_m+F A_0$ and $C_0=(\gamma - H A_0)/\alpha$. The dispersion is of capillary-gravity type. Indeed, the phase speed attains its maximum at $k_c=\sqrt{\left( \,-1 + \sqrt{1+\alpha ^2(C_0-u_0)/\beta }\,\right) /\alpha }$ where $dC/dk=0$. For $0\le k<k_c$, waves behave dispersively as capillary ($dC/dk>0$) since shorter waves travel faster than longer waves, i.e. $C(k)-u_0\sim k^2$. For $k>k_c$, the dispersion changes to gravity type with longer waves that travel faster than shorter waves ($dC/dk<0$). When fifth-order dispersion vanishes, i.e. $\beta$ tends to zero, the capillary brunch extends to large wavenumbers since $k_c$ tends to infinity and the gravity dispersion brunch disappears. In this case, the phase speed tends asymptotically to $C\rightarrow C_0$ at large *k*’s.

Such a wave dispersion is associated to a hidden *elastic energy* that has a counterpart in axisymmetric Navier–Stokes flows, which are essentially two-dimensional (2-D) since vortex stretching is absent. To understand the physical origin of such a wave dispersion, Fedele and Dutykh^18^ consider the 2-D Euler equations for an inviscid fluid over the domain *V* in a Cartesian frame. Given the streamfunction $\psi$, the divergent-free velocity field $\textbf{v} = \left( -\partial _y\psi , \partial _x\psi \right)$ and the vorticity $\omega _z= \partial _{xx}\psi +\partial _{yy}\psi$. The equation of motion follows as

3 $$\begin{aligned} \partial _t\omega _z = -\textbf{v}\cdot \nabla \omega _z = -[\psi ,\omega _z], \end{aligned}$$

where the commutator $[f,g] = \partial _x f \partial _y g - \partial _y f \partial _x g$. Drawing on Morrison^24^, the Hamiltonian form follows as

4 $$\begin{aligned} \partial _t\omega _z = \left\{ \omega _z,{\mathscr {H}}\right\} , \end{aligned}$$

where

5 $$\begin{aligned} {\mathscr {H}} = \frac{1}{2}\int _{V}\left| \nabla \psi \right| ^{2}\;dV = -\frac{1}{2}\int _{V}\omega _z\psi \; dV \end{aligned}$$

is the kinetic energy, or Hamiltonian of the system and the non-canonical Poisson brackets are defined as

6 $$\begin{aligned} \left\{ F,\; G\right\} = \int _{V}\omega _z\left[ \frac{\delta F}{\delta \omega _z},\frac{\delta G}{\delta \omega _z}\right] \;dV, \end{aligned}$$

where $\delta$ denotes variational derivative. Energy is conserved as ${\mathscr {H}}$ is an invariant of motion. Such a Hamiltonian structure naturally lend itself to a physical analogy between the fluid motion and the deformation of an elastic membrane. Indeed, the Hamiltonian ${\mathscr {H}}$ can be interpreted as the elastic energy of a thin membrane subject to tensional forces. The surface $\psi (x,y,t)$ represents the time-varying field of vertical displacements of the deformed membrane and the vorticity $\omega _z$ is proportional to the mean curvature $\kappa$ of the surface $\psi$. For a given elastic energy ${\mathscr {H}}$ of the membrane, curvature $\kappa$ changes over space and time according to Eq. (4). The membrane surface locally bends sharply if $\kappa$ increases, or flattens if $\kappa$ decreases. Since fluid streamlines are the contours of the streamfunction $\psi$, this implies that vortices intensify or attenuate where the surface curvature $\kappa$ is high or low.

In summary, the theoretical phase speed in Eq. 2 predicts that capillary-type dispersion is active in the inertial range of axisymmetric flow turbulence. In the following, we will investigate the dispersive properties of spanwise and streamwise vorticity of turbulent channel flows in comparison to the theoretical CH dispersion in Eq. (2).

**Figure 2:**
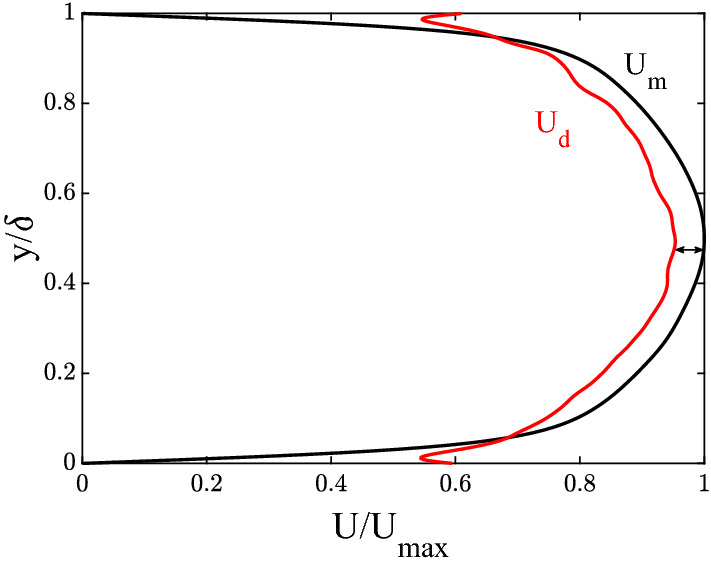
Mean velocity profile $U_m(y)$ (black line) and mean dynamical, or Taylor, velocity profile $U_d(y)$ (red line).

## Turbulence in channel flows

Consider an incompressible three-dimensional (3D) flow $U(x,y,z,t)=(u,v,w)$ in a channel of rectangular cross-section. The horizontal *x*-axis is in the streamwise direction of negative pressure gradients. The vertical *y*-axis is in the wall-normal direction pointing from the lower to the upper wall. Finally, the spanwise *z*-axis is chosen so that (*x*, *y*, *z*) is an orthonormal coordinate system. The channel height is $L_y=2 \delta$, the streamwise length is $L_x=4\pi \delta$ and the width $L_z=2 \pi \delta$ with $\delta$ the half channel height. At the bottom $y=0$ and at the top of the wall $y= 2 \delta$. The flow in periodic in both streamwise and spanwise directions. We shall investigate numerically the case of a turbulent flow at the bulk Reynolds number $\textsf{Re}_b=5600$, or the centerline Reynolds number $\textsf{Re}_c=3300$, and the relevant flow parameters are described in the “Methods” section. The simulated turbulent flow is generally characterized by a chaotic motion of vortices advected in an irregular and space-time varying flow field. In particular, Fig. 1 depicts vortices in the simulated field being mainly advected by the mean flow. A closer look at the flow dynamics reveals that the (encircled) vortical structure created at the wall is advected and lifted up near the centre of the channel (related animation Movie S1). In a reference frame moving with the vortex, it is evident that the flow structure, while being advected by the mean flow, changes its shape continuously by focusing and defocusing its energy content through a modulation of its vorticity. Since we want to investigate the dispersive character of turbulence, without loosing generality and to easily manage the computational complexity of the data analysis, we will study spanwise averages of vorticity and velocity fields. Such an average does not annihilate the dispersive features of the flow as discussed below.

## Beyond Taylor’s hypothesis

In channel flow turbulence vortical patches breath and deform while being advected downstream by the mean flow. In this case, the velocity *U* of a vortical structure can be decomposed as^2^

7 $$\begin{aligned} U=U_{d}+U_{g}, \end{aligned}$$

where the dynamical velocity $U_{d}$, or Taylor speed^1^, relates to the inertia of the turbulent flow and to the external forcing (e.g. pressure gradients), and $U_{g}$ is the geometric velocity induced by the deformation of vortices^2^. When turbulence is weak, vortices are transported passively at the mean flow speed $U_m$, i.e. $U_d \approx U_m$, and the geometric component is negligible^2,3^. When turbulence is not weak, Fedele et al.^2^ showed that the geometric velocity $U_{g}$ is significant and it is induced by the shape-changing dynamics of vortices. In particular, consider the spanwise vorticity field $\omega _z$. According to Taylor’s hypothesis^1^, turbulent flow is filled by frozen vortices advected downstream with the dynamical velocity^2,3^

8 $$\begin{aligned} U_{d}(y,t)= -\frac{\langle \partial _t \omega _z\partial _x \omega _z\rangle _{x,z} }{\langle \partial _x \omega _z \partial _x \omega _z\rangle _{x,z}}, \end{aligned}$$

where brackets $\langle \cdot \rangle _{x,z}$ denote space average in the *x* and *z* directions and $\partial _t$ and $\partial _x$ denote time and space differentiation. In our study case, turbulence is not weak and $U_m$ and $U_d$ differ as shown in Fig. 2. Note that the dynamical velocity $U_d$ does not tend to zero at the wall because vorticity and its temporal and spatial gradients do not vanish at the wall. Similar trend is also observed in the experiments by Fedele et al.^2^. The excess mean velocity $U_m-U_{d}$ is the geometric component $U_g$, which is not negligible since the turbulent fluctuations are not weak. Thus, vortices are advected at a speed different than the Taylor, or dynamical speed $U_d$^3^. Fedele et al.^2^ showed that their shape changing dynamics induces an additional self-propulsion velocity, or geometric speed $U_g$. A large excess of geometric velocity implies a strong regime of turbulence and Taylor’s hypothesis is not satisfied at all^2,3^.

In the following, we will show that the occurrence of geometric velocities is the kinematic manifestation of a hidden ocean-like dispersion of turbulence in analogy with surface gravity waves. To do so, we first briefly overview the dispersive properties of ocean wave groups and then investigate the ocean-like dispersion of the analogous wave groups, or packets of spanwise and streamwise vorticity.

## Slowdown and speedup of dispersive surface gravity waves

In a typical oceanic sea state, when the wind blows on an initially flat sea surface it generates first capillary waves, i.e. short waves (from about a few millimetres and up to a couple of centimetres) whose propagation is dominated by surface tension. Though small, under the action of the wind, capillary waves tends to group and grow in size, both in wavelength and amplitude, originating waves for which gravity acceleration dominates their propagation. The two restoring mechanisms of surface tension and gravity determine their duration span. A capillary wave soon flattens as the wind stops blowing, while a gravity wave, once formed by the wind, continues its propagation for long time in the form of a swell even without any forcing wind. Gravity and capillary waves manifest different dispersion properties. The dispersion relation, or dependence of the wave frequency $\omega (k)$ from the wavenumber *k*, characterizes the two different type of waves. In particular, for capillary waves $\omega \sim k^{3/2}$, while for gravity waves in deep water $\omega \sim k^{1/2}$. In the former case, the phase speed of the wave is $C=\omega /k$, and shorter capillary waves travel faster than longer capillary waves ($C\sim k^{1/2}$). On the contrary, shorter gravity waves tend to travel slower than longer gravity waves ($C\sim k^{-1/2}$).

Ocean waves typically travel in groups, or packets, exhibiting a complex propagation during focusing^8,14^. The speed of the largest crest of an unsteady wave group is different than the phase speed^9,10^. For example, the largest crest of a group traveling in deep waters slows down as it attains its maximum amplitude at focus and then speeds up in the following decaying phase. On the contrary, crests of capillary wave packets first speeds up while they reach their peak and then slow down^10^. The slowdown or speedup of crests is the kinematic manifestation of the natural dispersion of surface gravity waves. Similarly to the speed of vortical patches in Eq. 7, Fedele^9^ showed that the crest speed of the largest wave of a group traveling along the *x* direction can be decomposed as the sum of a dynamical velocity, or phase speed, and a geometric component that depends on the shape-changing dynamics of the crest

9 $$\begin{aligned} C_{crest}=C_{d}+C_{g}, \end{aligned}$$

and the dynamical velocity follows as^9^

10 $$\begin{aligned} C_{d}= -\frac{\langle \partial _t \eta\, \partial _x \eta \rangle _x }{\langle \partial _x \eta\,\partial _x \eta \rangle _x}, \end{aligned}$$

where $\eta (x,t)$ is the vertical displacement of the wave surface from the mean sea level, and brackets $\langle \cdot \rangle _x$ denote space average. Fedele^9^ showed that $C_d$ is close to the phase speed $C_0=\omega _0/k_0$, where $k_0$ is the wavenumber at the spectral peak and $\omega _0=\omega (k_0)$ is the associated frequency that follows via the dispersion relation. Wave packets containing energy in a broad range of wavenumbers manifest greater dispersion and their crests change shape significantly during focusing, while slowing down or speeding up^10^. As a result, in broadband wave packets the crest speed differs from the phase speed $C_0$ by the geometric component $C_g$, as the manifestation of the dispersion experienced by the wave group. On the contrary, narrowband wave packets with energy distributed in a small range of wavenumbers are weakly dispersive. Thus, they do not exhibit significant speedup/slowdown and $C\approx C_0$^10^, or equivalently $C_g\approx 0$.

The dispersion properties of surface gravity wave groups are now further explored by way of an analytical approach. Without loosing generality we consider a generic one-dimensional (1-D) long-crested wave packet traveling along the *x* direction, whose surface displacements from the mean sea level is $\eta (x,t)$. Our results also extend to two-dimensional (2-D) short-crested wave packets^10^. We rescale space and time as dimensionless, i.e. $x\rightarrow k_0x$ and $t\rightarrow \omega _0 t$. Assume a generic dispersion relation $\omega (k)$ and the packet energy is distributed in wavenumber space in accord with an amplitude spectrum *A*(*k*), where *k* is normalized by $k_0$ and $\omega$ by the corresponding frequency $\omega _0$ at the spectral peak. Drawing on Fedele et al.^10^

11 $$\begin{aligned} \eta (x,t)=h\int _{-\infty }^{\infty } A(k)\exp \left[ i\left( k x-\omega (k) t+\alpha \theta (k)\right) \right] dk, \end{aligned}$$

where $\alpha$ is the defocussing factor with values in [0, 1] and $\theta (k)$ is a random phase function of *k*. In particular, at any *k*, the phases are independent and uniform random variables distributed in $[-\pi , \pi ]$. When $\alpha =0$, perfect focusing occurs at the point $x= 0$ at time $t= 0$. Here, the tallest wave in the group reaches its maximum crest height *h* as a result of perfect phase coherence leading to a constructive superposition of the elementary harmonic waves whose amplitudes depend on the packet amplitude spectrum *A*(*k*). For $\alpha >0$, a degree of phase decoherence leads to an imperfect focusing and the maximum height at focus reduces to^10^ $h \sin (\pi \alpha )/(\pi \alpha )<h$. Fedele et al.^10^ demonstrated that wave packets with dispersion $\omega (k)\sim k^n$ ($n>0$) exhibit crest speedup for $n>1$ ($n=3/2$ for capillary waves), while a crest slowdown occurs for $0<n<1$ ($n=1/2$ for deep-water gravity waves).

Consider now the dispersion law $\omega =k^2$ of capillary-type waves. If the wave spectrum is Gaussian-shaped

12 $$\begin{aligned} A(k)=\frac{1}{\sqrt{2 \pi s^2}}e^{-\frac{(k-1)^2}{2 s^2}} \end{aligned}$$

with spectral bandwidth *s*, the Fourier integral in Eq. (11) can be solved analytically for $\alpha =0$ and the surface displacements of a perfect focusing group follows as

13 $$\begin{aligned} \eta (x,t)=\frac{1}{\sqrt{1+2 i s^2 t}}\exp \left\{ -\frac{1}{2s^2}\left[ 1-\frac{(1+i s^2x)^2}{1+2 i s^2 t}\right] \right\} . \end{aligned}$$

From Eq. (11), a degree of phase decoherence ($0<\alpha <1$) is introduced by convolving $\eta (x,t)$ with the stochastic defocussing kernel $f(x;\alpha )$, i.e. $(\eta *f)(x)$, where

14 $$\begin{aligned} f(x,\alpha )=\int _{-\infty }^{\infty }\exp \left[ i\left( k x+\alpha \theta (k)\right) \right] dk. \end{aligned}$$

By flipping space with time $(x=-t,t=-x)$ we get the surface displacement $\eta (-t,-x)$ of a deep-water gravity wave packet with dispersion $k=\omega ^2$, or $\omega =\sqrt{k}$.

**Figure 3:**
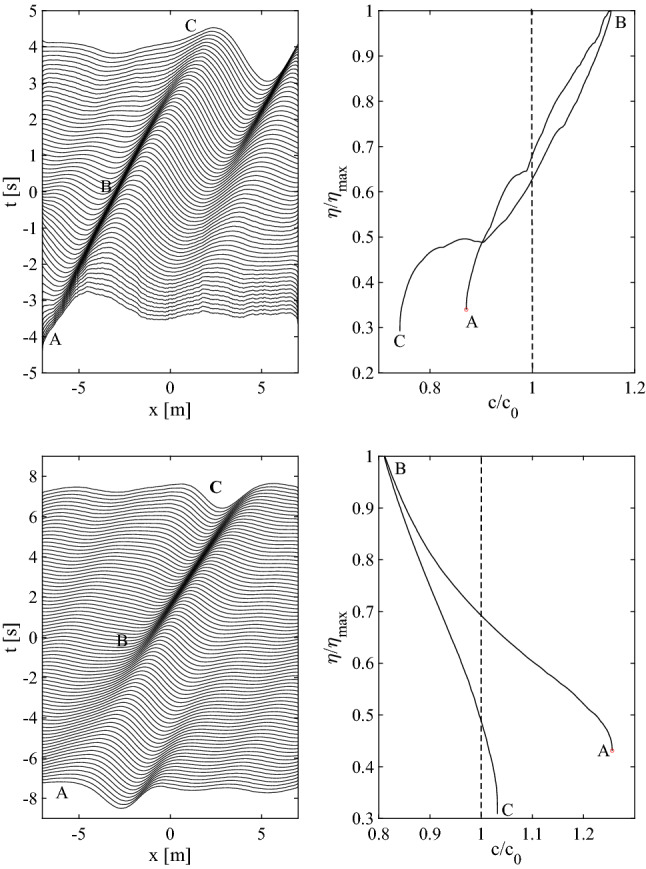
Kinematics of a Gaussian capillary wave group (top panels) and of a gravity wave group in deep waters (bottom panels): (left) space-time evolution of the wave elevation $\eta$, (right) hysteresis curve of the crest height $\eta /\eta _{max}$ as a function of the crest speed $C/C_0$, where $\eta _{max}$ is the maximum crest height and $C_0$ is the phase speed. The focusing is imperfect and the wave evolution before and after focus is asymmetric. Waves travel from left to right.

The space-time evolution of the wave surface displacements of a Gaussian capillary wave group (spectral bandwidth $s=0.3$, defocussing $\alpha =0.1$) is shown in the top-left panel of Fig. 3. The top-right panel depicts the crest height $\eta /\eta _{max}$ versus the crest speed $C/C_0$, where $C_0$ is the phase speed at the spectral peak and $\eta _{max}$ is the maximum height attained. From the left panel three essential phases are noticeable: a speed-up phase (A) before focus, with the maximum crest speed attained at the focusing (B), followed by a slowdown (C) after focus. The shape changing of the crest is the result of longer waves being ahead of shorter waves before focus at (A). These reach the longer waves at the focus time in (B) and surpass them after focus (C). In the initial phase the crest leans backward, it becomes symmetric at focus, and then leans forward after. Note that the speedup and slowdown phases are not symmetric because the focusing is imperfect ($\eta _{max}\approx 0.98 h$). Thus, the crest speed evolution does show hysteresis in the top-right panel. Note that the ratio $C/C_0$ at focus (B) is close to 1.10. Thus, the speedup is $10\%$ of the phase speed $C_0$ and equals the geometric velocity $C_g$ resulting in significant changes of the crest shape during the wave motion. Due to wave dispersion, small-scale waves travel faster than large-scale waves ahead of them, which are surpassed after the focusing point where the large peak occurs.

The kinematics of an imperfect focusing of a Gaussian wave group in deep waters (spectral bandwidth $s=0.3$, defocussing $\alpha =0.1$) is depicted in the two bottom panels of the same Fig. 3. Here, in the left panel we observe an initial slowdown of the crest before focus (A), with the minimum crest speed attained at focusing (B), followed by a phase of speeding-up (C). The crest shape-changing here is the result of the longer waves being behind the shorter waves before focusing at (A), then they reach the shorter waves at focus in (B) and surpass them after focus (C). In this case, the crest kinematics is anti-symmetric to that of the capillary wave group (see top panels of the same Figure). Indeed, before focus the crest leans forward, it shapes symmetrically at focus, and then leans backward after. From the bottom-right panel, the ratio $C/C_0$ at focus (B) is close to 0.8, indicating a $20\%$ slowdown of the crest speed, and the geometric component $C_g\approx -0.2 C_0$. Note that small-scale waves travel slower than large-scale waves behind them, the former being surpassed after the focusing point where the large peak occurs.

The observed shape changing of the wave crest is the kinematic manifestation of the dispersion of the wave group. The crest slowdown or speedup phenomenon occurs when unsteadiness plays a role in the wave group propagation, and it has its theoretical origin in the geometric phase framework in quantum mechanics^9,25^. Note that our results and conclusions are also valid for two-dimensional (2-D) short-crested wave packets as shown by Fedele et al.^10^. Nonlinearities make surface gravity waves less dispersive and the effects of slowdown or speedup of the dominant crest is slightly reduced, but still significant^10,26^.

### Quasi-determinism of waves

Waves of an oceanic sea state are typically modeled as realizations of an homogeneous and stationary Gaussian sea^27^. Consider a two-dimensional (2-D) wave field of surface displacement $\eta (\textbf{X},t)$ and position vector $\textbf{X}=(x,y)$. The space-time structure of the wave field surrounding a very large crest height, or surface maximum *h* that occurred at the point $\mathbf {X_0}=(x_0,y_0)$ at time $t_0$, can be predicted almost surely as $h\rightarrow \infty$. In simple words, the number of realizations of a Gaussian sea surrounding a very large wave crest are limited. The wave field can only develop downward from the large peak, and in the limit of infinite height *h*, with probability tending to one it assumes the quasi-deterministic (QD) form given by conditional mean^27–29^

15 $$\begin{aligned} \eta _{QD}(\textbf{X}+\mathbf {X_0},t+t_0)=\overline{\eta (\textbf{X} +\mathbf {X_0},t+t_0)|\eta (\mathbf {X_0},t_0)=h)}=h\, \frac{\langle \eta (\textbf{X}+\mathbf {X_0},t+t_0) \eta (\mathbf {X_0},t_0) \rangle }{\langle \eta (\mathbf {X_0},t_0)^2 \rangle }, \end{aligned}$$

where $\overline{\left( \cdot \right) }$ denotes ensemble average and brackets $\langle \cdot \rangle$ denote time average. The cross-correlation between the surface displacement at the focusing point and that at the surrounding points encodes the wave structure around the extreme maximum. We note that the QD model is valid for nonstationary and nonhomogeneous fields and it has been applied to nonlinear fields^29,30^. For one-dimensional (1-D) wave fields and applications discussed below

16 $$\begin{aligned} \eta _{QD}(x+x_0,t+t_0)=\overline{\eta (x+x_0,t+t_0)|\eta (x_0,t_0)=h)} = h\, \frac{\langle \eta (x+x_0,t+t_0) \eta (x_0,t_0) \rangle }{\langle \eta (x_0,t_0)^2 \rangle }, \end{aligned}$$

where $\eta (x,t)$ is the surface displacement. QD theory^27–29^ is very general as Eq. (15) also applies to three  (3-D) and higher dimensional random fields. In our studies, without losing generality we only focus on 2-D fields. We note in passing that Schmidt and Schmid^31^ formulated a Proper Orthogonal Decomposition method based on the space-time correlation of turbulent measurements. Such an approach is then applied to estimate conditional averages and study the structure of turbulent flows near maxima. Here, we wish to emphasize that the space-time correlation in Eq. (15) already encodes the turbulent structure around maxima^27–29^.

## Results: wave dispersion of turbulence

In this section we will investigate the dispersive character of channel turbulence. To bear the high computational cost of handling tera-scale data set at hand, without loosing generality we will consider spanwise *z*-averages of vorticity and velocity fields. Such a spanwise average does not annihilate any wave dispersion of the flow. We are interested in the large-amplitude vortical structures analogous to the large wave groups of oceanic seas^10,29^. Unlike the small-amplitude vortical structures that occur sparsely over space and time, large-amplitude packets are isolated events in space-time. Thus, the 2D spanwise average neither alters their space-time evolution, nor their dispersive properties as supported by the following theoretical argument. Consider a large generic 3D dispersive wave packet field $\eta$ advecting along the streamwise direction *x*. According to QD theory^27–29^, the wave field is represented by the Fourier integral

$$\begin{aligned} \eta (x,y,z,t)=\int \int \int S(k_{x},k_{y},k_{z})\exp \left[ i\left( k_{x}x+k_{y}y +k_{z}z-\omega (k)t\right) \right] dk_{x}dk_{y}dk_{z}, \end{aligned}$$

where $S(k_{x},k_{y},k_{z})$ is the spectrum, or Inverse Fourier transform of the covariance of the wave field and the dispersion relation $\omega =U k_x+\alpha \,(k_x^2+k_y^2+k_z^2)$ includes a dispersionless component (streamwise advection at the speed *U* along *x*) and a capillary-type component ruled by the parameter $\alpha$. Averaging along the homogeneous spanwise direction *z* over a length $L_z$ yields the 2D wave field

$$\begin{aligned} \left\langle \eta \right\rangle _{z}=\int _{-L_{z}/2}^{L_{z}/2}\eta \, dz=\int \int \widetilde{S}(k_{x},k_{y},t)\exp \left[ i\left( k_{x}x+k_{y}y -\widetilde{\omega }(k_{x},k_{y})t\right) \right] dk_{x}dk_{y}. \end{aligned}$$

This averaged field still exhibits capillary-type dispersion and streamwise advection as $\widetilde{\omega }(k_{x},k_{y})=U k_x+ \alpha \,(k_{x}^{2}+k_{y}^{2})$ and the associated 2D spectrum

$$\begin{aligned} \widetilde{S}(k_{x},k_{y},t)=\int S(k_{x},k_{y},k_{z}) \frac{\sin \left( k_{z}L_{z}/2\right) }{k_{z}L_z/2}\exp \left( -i\alpha k_{z}^{2}t\right) dk_{z}. \end{aligned}$$

For example, consider the wave packet field $\eta$ propagating along the streamwise direction (peak wavenumber $k_0$) with Gaussian-shaped spectrum

$$\begin{aligned} S(k_{x},k_{y},k_{z})\sim \frac{1}{(2\pi )^{3/2}s_{x}s_{y}s_{z}}\exp \left( -\frac{(k_x-k_0)^2}{2s_x^2}-\frac{k_y^2}{2s_{y}^{2}}-\frac{k_{z}^{2}}{2s_{z}^{2}}\right) , \end{aligned}$$

where $s_x,s_y,s_z$ are spectral bandwidths in the streamwise, vertical and spanwise directions, respectively. Then,

$$\begin{aligned} \widetilde{S}(k_{x},k_{y},t)\sim \frac{\exp \left( -\frac{k_{x}^{2}}{2s_{x}^{2}} -\frac{k_{y}^{2}}{2s_{y}^{2}}\right) }{2\pi s_{x}s_{y}}\left| F(t)\right| \exp \left[ i\varphi (t)\right] , \end{aligned}$$

where $\varphi (t)=\arctan [\textrm{Im}F(t)/\textrm{Re}F(t)]$ is the phase of the complex function

$$\begin{aligned} F(t)=\frac{\sqrt{2\pi }}{Ls_{z}}\textrm{Erf}\left( \frac{s^{2}}{2\sqrt{2}\sqrt{1+2s^{2}i\,\alpha t}}\right) , \end{aligned}$$

where $\textrm{Erf}(z)=2/\sqrt{2\pi }\int _{0}^{z}\textrm{e}^{-w^2}dw$ is the error function. Note that *F*(*t*) simply modulates the spectrum in time. The phase $\varphi (t)$ is independent of the wavenumbers $k_x,k_y$ and both advection and capillary-type dispersion are preserved by the spanwise averaged field. Indeed, the frequencies $\widetilde{\omega }(k_{x},k_{y})$ are simply shifted by the wavenumber-independent shift $\Delta \omega (t)=-d\varphi /dt$. A similar argument also holds for gravity-wave dispersion. So, if 2D spanwise averages of a 3D turbulent field are dispersive, so is their parent 3D field. We note in passing that the aforementioned approach has similarities to the covariant migration technique in radar imaging^32^. Here, (*x*, *y*)-slice measurements of a (*x*, *y*, *z*)-wave field allows extrapolating along the depth *z* from the knowledge of the full wave dispersion.

It is also relevant to study the dispersive properties of spanwise vorticity of 3D incompressible turbulent fields, where stretching and tilting are directly connected to vortex deformations given by the conservation of the angular momentum and induced by gradient of velocities aligned with the vorticity vector. Although space averages partially smooth out vortex dynamics, the information content about vortex deformations is unaltered by the spanwise averaged vorticity values in the *xy* plane. The lengthening or shortening of the filaments in the spanwise *z* direction corresponds to an increase or decrease in size of the vortex patch. Since streaks extend mainly in the streamwise direction, 3D turbulence is mainly generated by the action of the spanwise vortex stretching mechanism at Reynold number $Re_{\tau } = 180$ (see “Methods” section for parameter definition).

### Spanwise vorticity

Consider the fluctuations of the mean spanwise vorticity field $\overline{\omega }_z(x,y,t)=\langle \omega _z(x,y,z,t)\rangle _z$, where brackets denote space average in the spanwise direction *z*. We note that the core of the simulated turbulent field is quasi-Gaussian, with spots of high kurtosis $\sim 5$ punctuating the field, indicating the expected intermittency at the small scales. We use Eq. (16) and explore the dispersion features of turbulence by applying Boccotti’s QD theory^27,29^. To account for the non-homogeneity of the vorticity field in space, we apply QD theory to the horizontal cross-sectional vorticity field $\widetilde{\omega }_z(x,t)=\overline{\omega }_z(x,y=y_0,t)$ at a prescribed distance of $y=y_0$ from the wall. We will consider indicative values of $y_0$ near the wall ($y^+ = 0.027$) and near the channel’s center ($y^+=160.2$), with the center at $y^+=177.8$. Variations around those positions yield the same conclusions. The vorticity field is analogous to the free-surface elevation $\eta$ of an oceanic wave field, and we are interested in the most probable space-time turbulent structure around peaks of vorticity. Such a structure resembles a wave group that we refer to as a QD vortical packet.

**Figure 4:**
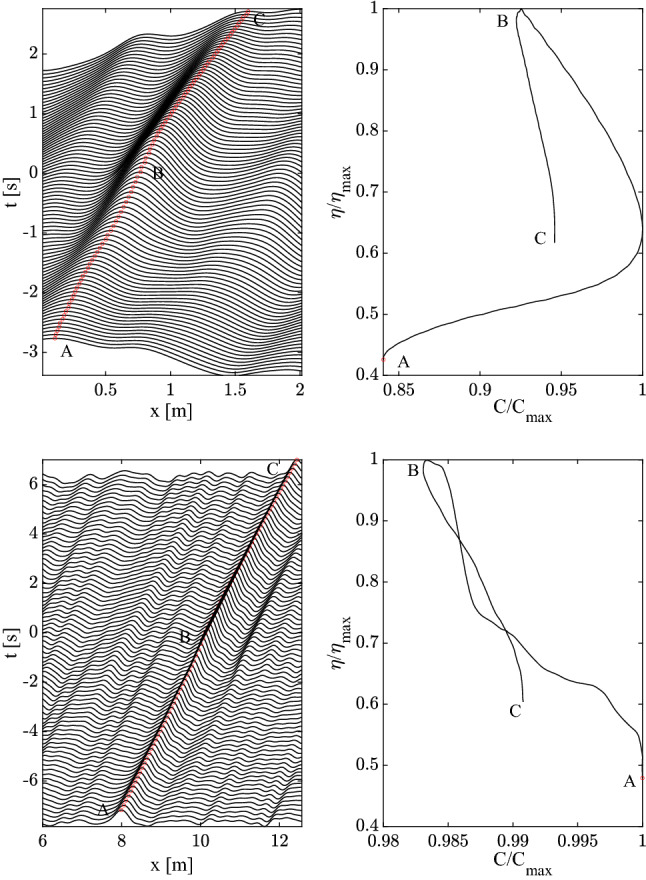
Kinematics of QD spanwise vortical packets: space-time evolution of a QD vortical packet near the wall (left-top panel) and near the channel’s centre (left-bottom panel) of amplitude $\eta$. Right panels depict the corresponding hysteresis curve of the crest maximum $\eta /\eta _{max}$ as a function of the crest speed $C/C_{max}$, where $\eta _{max}$ and $C_{max}$ are the maxima of amplitude and phase speed. The QD packet behaves as a capillary wave-like group near the wall and as as deep-water wave group near the channel’s centre (bottom panel). Waves travel from left to right.

The space-time evolution of a QD spanwise vortical packet near the wall is estimated from Eq. (16) and shown in the top panels of Fig. 4. In particular, the left panel depicts the vorticity fluctuations along the streamwise direction *x* at successive instants of time. We observe an asymmetric evolution of the packet growing in amplitude as it reaches its maximum peak followed by a decaying phase. The right panel depicts an hysteresis of the peak, or crest speed as a function of its vorticity amplitude. This indicates that the packet evolution is asymmetric before and after the focus^10^. The hysteresis could be due to the nonlinear nature of the flow and imperfect focusing. Note that the space-time evolution of the QD vortical packet resembles that of a capillary-type wave packet (see top panels of Fig. 3): initially the packet speeds up (*A*-*B*) as it attains its maximum in *B* and then slows down asymmetrically (*B*-*C*). We observe a dispersive trend of gravity-type in the space-time evolution of the QD vortical packet near the centre of the channel, as depicted in the bottom panels of the same Fig. 4. The hysteresis shown in the right panel of the same figure indicates that the packet slows down as it reaches its peak, and then speeds up in the following decaying phase. So, the dispersive behaviour is similar to that of deep-water gravity waves (see also the bottom panels of Fig. 3).

Our analysis gives evidence that vorticity dynamics manifests wave-like dispersive properties that vary from the wall to the channel’s centre. The small-scale structures near the wall behave dispersively as capillary-type waves with a speedup phase before focus followed by a slowdown. On the contrary, the large-scale structures near the channel’s centre behave as deep-water waves as they slow down before reaching the focusing point and speed up after. This implies that in the boundary layer, initially dominated by small scales, the large scales become even more significant. The spanwise vorticity field near the channel’s centre, where gravity-type dispersion in the inertial range dominates over the capillary-type regime at the small scales, is analogous to the wave surface field of a mature oceanic sea. Here, the large-scale waves in the form of swells propagate purely to gravity dispersion and minor small-scale capillary waves on top of the swells are generated because of the local wind^33^. On the contrary, the vorticity field near the wall, where capillary-type dispersion dominates, is analogous to the wave field of a young sea, where the small waves generated by a forcing wind are capillary. As waves grow both in size an amplitude, eventually gravity effects will rule their propagation.

**Figure 5:**
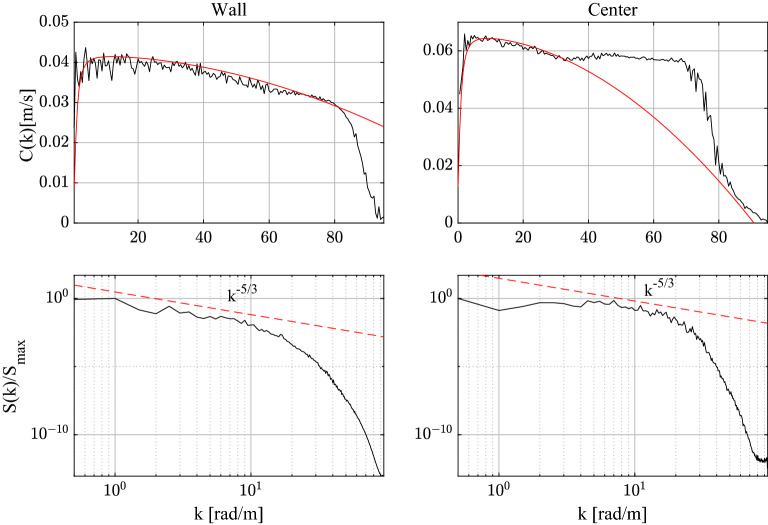
Spanwise vorticity: observed phase velocity *C*(*k*) of the field near the wall and the channel’s centre (black lines) and theoretical CH dispersion of Eq. (2) (red lines) are shown in the top panels. The respective spectra $S(k)/S_{max}$ normalized by their peak amplitude $S_{max}$ are shown in the bottom panels. Estimated parameters $u_0,C_0,\alpha , \beta$ of the CH dispersion are also shown. Wall: $u_0=0.001$, $C_0=0.042$, $\alpha =0.95$, $\beta =1.9\cdot 10^{-6}$. Center: $u_0=0.0001$, $C_0=0.0658$, $\alpha =0.95$, $\gamma =7.5\cdot 10^{-6}$.

The wave-like dispersion of the spanwise vorticity $\omega _z$ can be quantified by estimating the phase speed *C*(*k*) as a function of the wave number *k* as^3^

17 $$\begin{aligned} C(k)=\textrm{Im}\frac{\left\langle \Phi (k,t)\,\partial _{t}\Phi ^{*}(k,t)\right\rangle }{\left\langle \Phi(k,t)\,\Phi ^{*}(k,t)\right\rangle k}, \end{aligned}$$

where $\Phi (k,t)$ is the Fourier spectrum of the spanwise vorticity field $\widetilde{\omega }_z(x,t)$ and brackets $\langle \cdot \rangle$ denote time average. The top panels of Fig. 5 show the observed *C*(*k*) near the wall and at the channel’s centre. The respective spectra *S*(*k*) are also shown in the bottom panels. We note that the observed wave dispersion of the spanwise vorticity fairly agrees with the theoretical CH dispersion of Eq. (2) indicating a capillary-gravity type dispersive behavior. Near the wall, capillarity is dominant at small wavenumbers, or large scales, where energy is mostly localized. As a result, the kinematics of the corresponding QD vorticity packet manifests a speed-up of capillary-type groups ($\omega \sim k^2$, see top panels of Fig. 4). Near the channel’s centre, the net effect of the competing capillary-gravity type dispersion is a mild dominance of gravity. A dispersionless branch ($\omega \sim k$) is also observed in the inertial range. In physical space, the space-time evolution of the corresponding QD vortical packet undergoes a mild slowdown typical of gravity wave groups (see bottom panels of Fig. 4).

**Figure 6:**
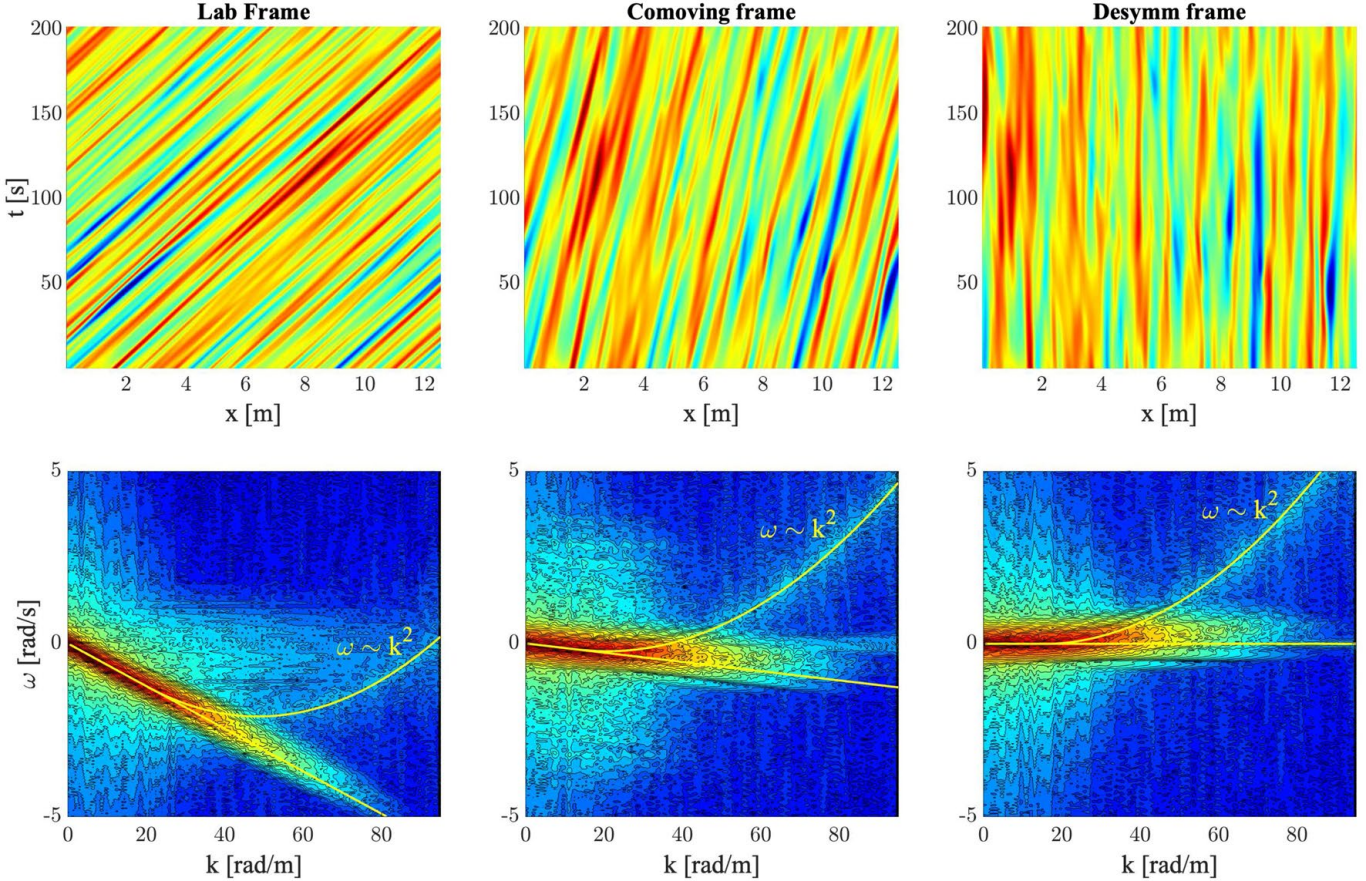
(Top panels) spanwise vorticity field $\omega _z(x,t)$ near the wall in the (left) laboratory, (centre) comoving and (right) desymmetrized frames. The respective wavenumber-frequency spectra are shown in the bottom panels.

The fluctuations of the spanwise vorticity field $\widetilde{\omega }_z(x,t)$ near the wall are shown in the top panels of Fig. 6 in three different reference systems: (left) fixed laboratory, (central) comoving and (right) desymmetrized frames^2,34^. The respective wavenumber-frequency spectra $S(k,\omega )$ are shown in the bottom panels of the same figure. In the laboratory frame the field is observed from the fixed Cartesian system used in the DNS simulations. Here, we note a drift of the wavy fluctuations of spanwise vorticity due to the inertia of the flow advecting the vortical structures and to their shape-changing dynamics. The associated wavenumber-frequency spectrum indicates a capillary branch $\omega \sim k^{2}$ in agreement with the trend observed for the average frequency shown in Fig. 5. The same field observed in the comoving frame traveling at the dynamical velocity $U_d$ in Eq. (8) is shown in the central panel. Here, a residual drift, or shift is still present and it is induced by the deformation of vortices. Drawing on Fedele et al.^2^, the vorticity field observed in a desymmetrized frame traveling at the total speed $U_{d}+U_{g}$, which includes the geometric velocity, is shown in the right panel. The drift disappears and we can observe the pure deformation of the fluctuation field. Moreover, the spectrum has a clear capillary branch $\omega \sim k^{2}$ as observed in the laboratory and comoving frames, which was masked by the advection of the mean flow. This is in agreement with the capillary trend observed in the phase speed *C*(*k*) near the wall and depicted in the top-left panel of Fig. 5. In that figure, *C*(*k*) defined in Eq. (17) is an average over frequency of the phase speeds of the elementary harmonic waves composing the vorticity field with given wavenumber *k*.

We note that the dispersion of surface gravity waves is quenched as their wave amplitude increases because of nonlinear effects^10^, a precursor to breaking, or blowup^35^. Similarly, we expect that at larger Reynolds numbers nonlinearities will alter the observed wave dispersion, which then will also depend on the turbulent intensity of the flow.

**Figure 7:**
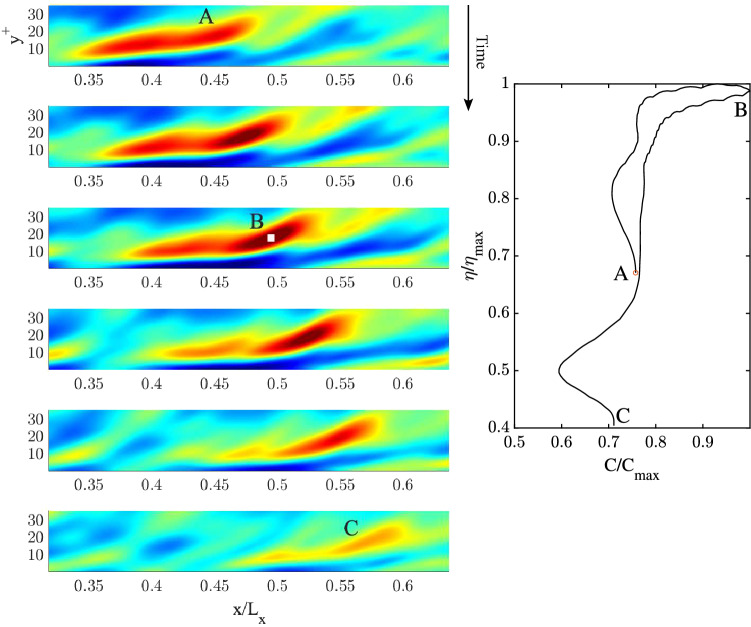
(Left) Space-time evolution of a 2D QD vortical packet of spanwise vorticity $\omega _z$ focusing near the wall at B ($y^+ =20.2$). Time increases from top to bottom, related animation Movie S2. (Right) hysteresis curve of the peak amplitude $\eta /\eta _{max}$ as a function of the peak speed $C/C_{max}$, where $\eta _{max}$ and $C_{max}$ are the vorticity and speed maxima. The area that extends to the centre of the channel ($y^+ =177.8$) is not shown to clearly depict the packet dynamics. Warmer (cooler) colors denote positive (negative) values of vorticity.

**Figure 8:**
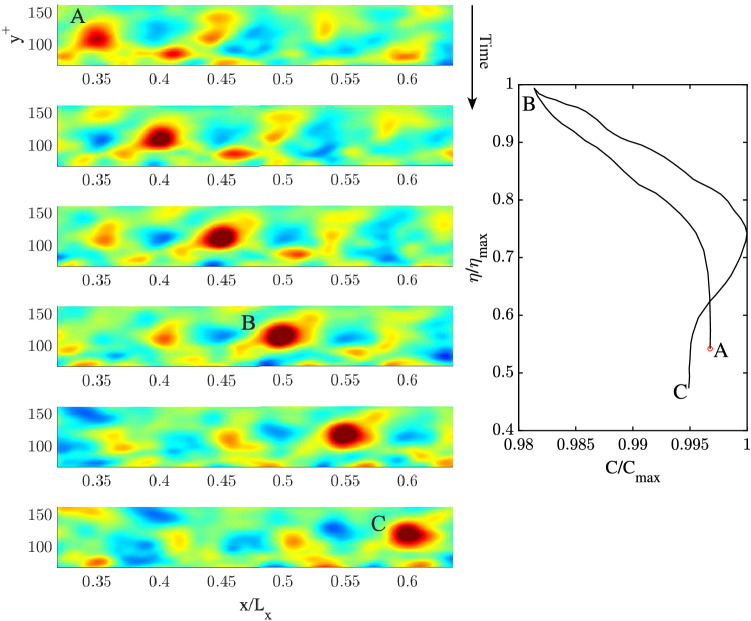
(Left) Space-time evolution of a 2D QD vortical packet of mean spanwise vorticity $\overline{\omega }_z(x,y,t)$ focusing near the centre of the channel at B ($y^+ =117.3$). Time increases from top to bottom, related animation Movie S3. (Right) hysteresis curve of the peak amplitude $\eta /\eta _{max}$ as a function of the peak speed $C/C_{max}$, where $\eta _{max}$ and $C_{max}$ are the vorticity and speed maxima. Warmer (cooler) colors denote positive (negative) values of vorticity.

### QD spanwise vortical packet structures

We wish to gain further insights into the space-time evolution of spanwise vorticity, deformation of vortices and their wave-like dispersive properties. To do so, we use Eq. (15) and apply Boccotti’s QD theory^27^ to the mean spanwise vorticity field $\overline{\omega }_z(x,y,t)$. The space-time evolution of the estimated QD vortical packet focusing near the wall ($y^+=20.2$) is depicted from top to bottom in the left panels of Fig. 7 (related animation Movie S2). To clearly depict the packet dynamics near the wall, the area that extends to the centre of the channel ($y^+=177.8$) is not shown. The QD packet travels from left to right along the *x* direction and attains its maximum at the focusing point at B. In particular, patches of vorticity generated at the wall are convected downstream (see first two panels from the top). During such motion the QD vortical packet consists of a large vortex patch advected downstream that splits into two vortices before focus. As the focus point is approached, the vortex ahead intensifies and speeds up more than the slower vortex behind suggesting a capillary-type dispersion. This is clearly observed in the hysteresis diagram of the speed of the large vortical patch shown in the right panel of the same figure. Both vortices dissipate after focus is attained. The vortex speed-up is in agreement with inviscid theory that predicts the vortex speed $U=\Gamma /L$, where $\Gamma$ is the circulation and *L* is the vortex width. As the focusing point is approached, the circulation of the large vortex increases more than its size resulting in a speedup. The left panel of Fig. 8 depicts the dynamics of the estimated QD vortical packet focusing near the centre of the channel  ($y^+=117.3$) from top to bottom (related animation Movie S3). The packet consists mainly of a large vortex patch advected downstream that splits into a very small vortex and into a large one before focus, which both dissipate after focusing is attained. No capillary-type dispersion is observed. The hysteresis diagram in the right panel of the same figure indicates a mild slowdown suggesting a gravity-type dispersion.

We note in passing that the 2D QD vortical packets are large vortices since their size $0.2<L<1.9\,\,\textrm{m}$ corresponds to wavenumbers $k=2\pi /L$ in the range $[3.3, 31.4]\,\,\mathrm {rad/m}$, where *L* is the streamwise length of the vortex. In that range, capillary-type dispersion is dominant near the wall, whereas near the centre the net effect is a mild dominance of gravity-type dispersion in agreement with the phase velocities *C*(*k*) depicted in the top panels of Fig. 5.

### Streamwise vorticity

**Figure 9:**
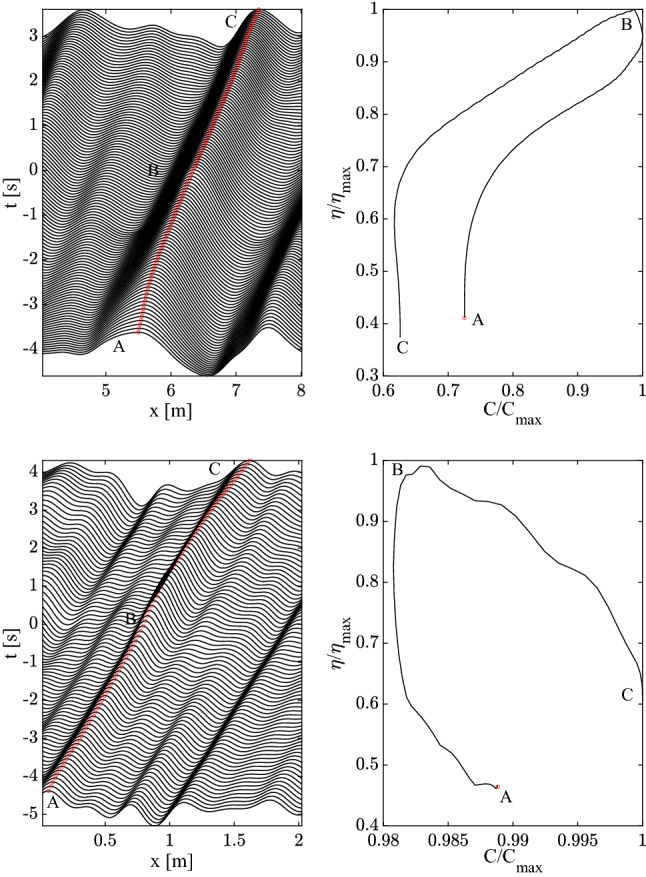
Kinematics of QD streamwise vortical packets: space-time evolution of a QD vortical packet near the wall (left-top panel) and near the channel’s centre (left-bottom panel) of amplitude $\eta$. Right panels depict the corresponding hysteresis curve of the crest maximum $\eta /\eta _{max}$ as a function of the crest speed $C/C_{max}$, where $\eta _{max}$ and $C_{max}$ are the maxima of amplitude and phase speed. The QD packet behaves as a capillary wave-like group near the wall and as deep-water wave group near the channel’s centre (bottom panel). Waves travel from left to right.

**Figure 10:**
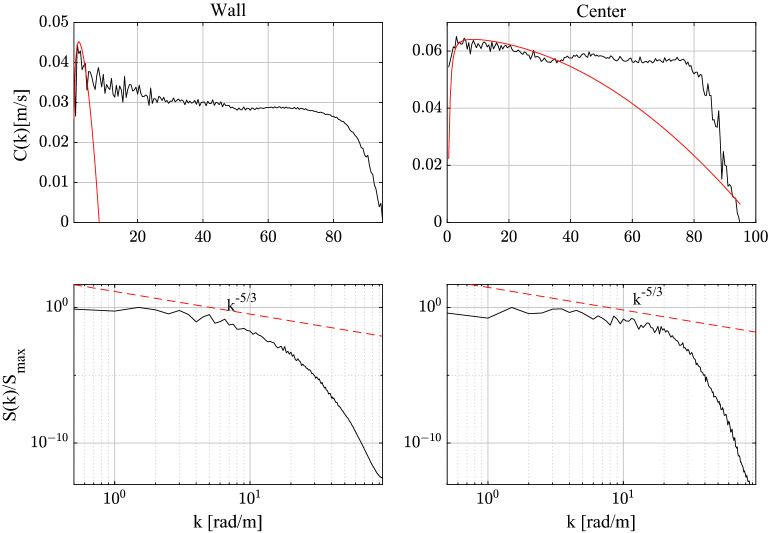
Streamwise vorticity: observed phase velocity *C*(*k*) of the field near the wall and the channel’s centre (black lines) and theoretical CH dispersion of Eq. (2) (red lines) are shown in the top panels. The respective spectra $S(k)/S_{max}$ normalized by their peak amplitude $S_{max}$ are shown in the bottom panels. Estimated parameters $u_0,C_0,\alpha , \beta$ of the CH dispersion are also shown. Wall: $u_0=0.001$, $C_0=0.051$, $\alpha =4$ $\beta =0.003$. Center: $u_0=0.001$, $C_0=0.065$,$\alpha =2$, $\beta =1.3 \cdot 10^{-5}$.

For completeness, we investigate the wave dispersion properties of the fluctuations of the mean streamwise vorticity field  $\overline{\omega }_x(x,y,t)=\langle \omega _x(x,y,z,t)\rangle _z$. The space-time evolution of the 1D QD vortical packet $\widetilde{\omega }_x(x,t)=\overline{\omega }_x(x,y=y^{+},t)$ near the wall ($y^+=0.027$) and channel’s centre ($y^+=160.2$) are shown in the left panels of Fig. 9. They indicate an asymmetric evolution of the packet growing in amplitude as it reaches its maximum peak followed by a decaying phase. The hysteresis of the crest speed (right panels of Fig. 9) confirms the capillary wave dispersion of turbulence near the wall and a mild gravity-type at the channel’s center. The observed phase velocity  *C*(*k*) depicted in the top panels of Fig. 10 is fairly explained by the theoretical CH dispersion of Eq. (2) at small wavenumbers, where energy is mostly localized.

**Figure 11:**
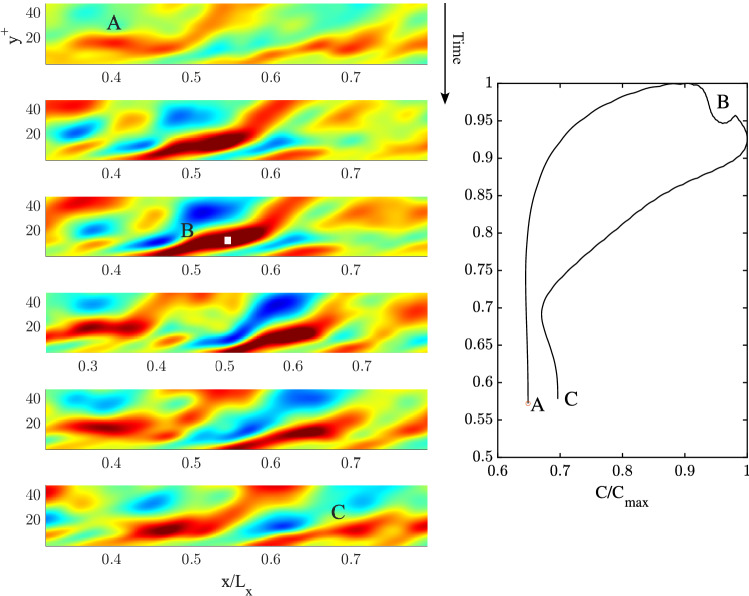
(Left) Space-time evolution of a 2D QD vortical packet of the mean streamwise vorticity $\overline{\omega }_x(x,y,t)$ focusing near the wall at B ($y^+ =13.2$). Time increases from top to bottom, related animation Movie S4. (Right) hysteresis curve of the peak amplitude $\eta /\eta _{max}$ as a function of the peak speed $C/C_{max}$, where $\eta _{max}$ and $C_{max}$ are the vorticity and speed maxima. Warmer (cooler) colors denote positive (negative) values of vorticity.

**Figure 12:**
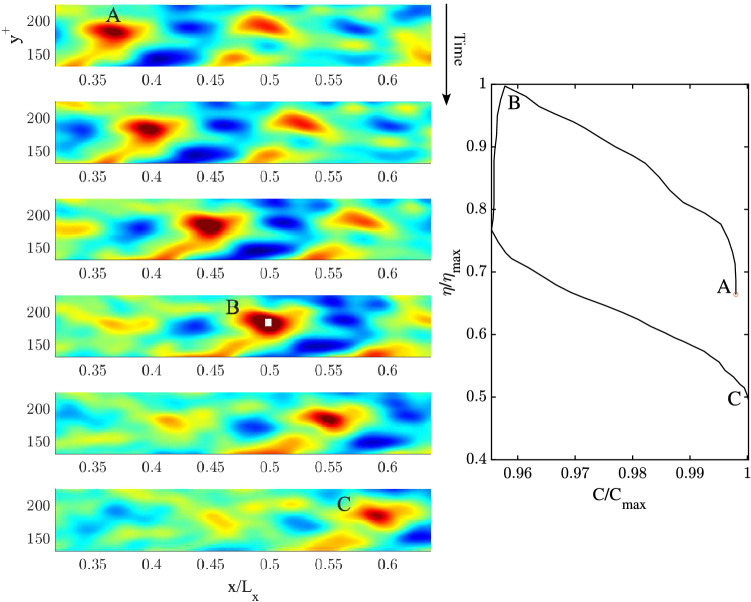
(Left) Space-time evolution of a 2D QD vortical packet of the mean streamwise vorticity $\overline{\omega }_x(x,y,t)$ focusing near the centre of the channel at B ($y^+ =177.8$). Time increases from top to bottom, related animation Movie S5. (Right) hysteresis curve of the peak amplitude $\eta /\eta _{max}$ as a function of the peak speed $C/C_{max}$, where $\eta _{max}$ and $C_{max}$ are the vorticity and speed maxima. Warmer (cooler) colors denote positive (negative) values of vorticity.

The space-time evolution of a QD vortical packet focusing near the wall ($y^+=13.2$) and its crest speed hysteresis are depicted in Fig. 11. The QD packet travels from left to right and focuses at B after speeding-up. Its intensification is the result of the interaction of two counter-rotating streamwise vortices. After focus, the two vortices dissipate while slowing down the packet indicating a capillary-type wave dispersion. Figure 12 depicts the space-time evolution of a QD vortical packet focusing at the channel’s centre ($y^+=177.8$). A large vortex patch is observed advecting downstream and no capillary-type dispersion is observed. The hysteresis diagram in the right panel of the same figure indicates a mild slowdown suggesting a gravity-type dispersion.

**Figure 13:**
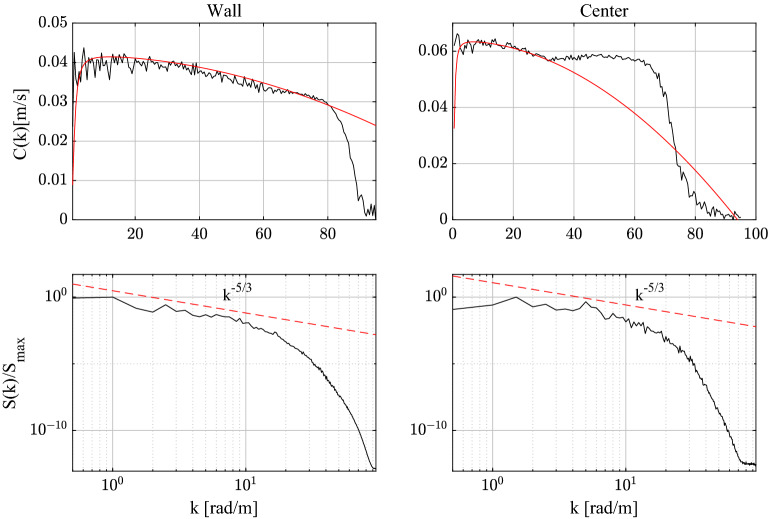
Streamwise velocity: observed phase speed *C*(*k*) of the field near the wall and the channel’s centre (black lines) and theoretical CH dispersion of Eq. (2) (red lines) are shown in the top panels. The respective spectra $S(k)/S_{max}$ normalized by their peak amplitude $S_{max}$ are shown in the bottom panels. Estimated parameters $u_0,C_0,\alpha , \beta$ of the CH dispersion are also shown. Wall: $u_0=0.001$, $C_0=0.042$, $\alpha =0.95$, $\beta =1.9 \cdot 10^{-6}$. Center: $u_0=0.001$, $C_0=0.064$, $\alpha =4$, $\beta =2.9 \cdot 10^{-5}$.

Finally, we note in passing that the theoretical CH dispersion of Eq. (2) fairly explains the phase speeds of the streamwise velocity field as shown in Fig. 13, suggesting a capillary-gravity dispersion of the large scale structures.

### Conclusions

Channel flow turbulence is studied by interpreting its vorticity as a random sea of ocean wave packet analogues. The wave-like properties of vortical packets is investigated by applying stochastic methods developed for random oceanic fields^27–29^. Our analysis of a moderate turbulent channel flow at the bulk Reynolds number $\textsf{Re}_b=5600$ provides evidence that the space-time evolution of spanwise and streamwise vorticity fields have strong analogy with the dispersive propagation of surface gravity waves. In particular, the small turbulent scales characterizing the flow near the wall behave dispersively like capillary waves, speeding up as they grow in intensity and then slowing down. On the contrary, large scales near the channel’s centre behave with a mild gravity dispersion typical of water waves in deep-waters. They tend to slowdown as they intensify and then to speedup. The observed deviations from Taylor’s hypothesis appear to be the signature of the dispersive nature of turbulence. Moreover, the observed wave dispersion fairly agrees with the theoretical dispersion predicted for axisymmetric flows^18,19,21^.

Similarly to surface gravity waves that behave less dispersively as their amplitude increases^10^, at larger Reynolds number flows we expect nonlinearities to alter wave dispersion, which thus has a dependence on the turbulent intensity of the flow. Finally, our approach to investigate dispersion properties of channel turbulence is general and can be extended to 3D fields.

## Methods

### Direct numerical simulations of channel flow turbulence

The developed turbulence in channel flow is a theoretical case consisting of a flow passing through two infinite parallel planes, driven by a constant pressure gradient. The physical parameters of the flow are the driving pressure gradient and the kinematic viscosity of the fluid $\nu$. The flow is characterized by its bulk Reynolds number $\textsf{Re}_b=\frac{U_b d}{\nu }$ based on the channel height $d=2\delta$ and the mean bulk velocity $U_b=\frac{1}{d}\int _{0}^{d} <u(x,y,z,t)>_{xz}\,dy$, where $< \cdot >_{xz}$ denotes space average in the *x* and *z* directions. We also define the centerline Reynolds number $\textsf{Re}_c=\frac{U_c \delta }{\nu }$ with $U_c$ the mean centerline velocity based on the half channel height $\delta$. The flow is laminar for $\textsf{Re}_c<1350$ and fully turbulent for $\textsf{Re}_c>1800$, although coexistence of laminar and turbulent states are evident up to $\textsf{Re}_c=3000$. The corresponding friction Reynolds number $\textsf{Re}_{\tau }=\frac{u_{\tau } d}{\nu }$ depends on the friction velocity $u_{\tau }$. We also define the dimensionless wall coordinate $y^+= y u_{\tau }/\nu$, where *y* is the vertical distance from the wall.

Direct numerical simulations (DNS) were carried out using the open-source software OpenFOAM^36^. The Navier–Stokes equations are numerically solved using the finite volume method and without any turbulence modeling of the unresolved smallest scales. This means that the whole range of simulated spatial and temporal scales are resolved. We carried out our simulations of a fully developed channel flow turbulence at the bulk Reynolds number $\textsf{Re}_b=5600$, or the centerline Reynolds number $\textsf{Re}_c=3300$ and the friction Reynolds number $\textsf{Re}_{\tau }=180$. We considered the same channel geometry as in Kim et al.^37^. In particular, the streamwise channel length is $L_x=4\pi \delta$, the height $L_y=d=2\delta$ and the width $L_z=2\pi \delta$, with $\delta =1\,\text {meter}$. We used a mesh grid with $(N_x=383)\times (N_y=192)\times (N_z=319)$ points, doubling the resolution of the mesh used in Kim et al.^37^. For more details on the numerical simulations and mesh convergence we refer to Pilloton^38^. In order to simulate a domain of infinite size, periodic boundary condition were applied in the streamwise and spanwise direction for the velocity and pressure fields. Otherwise, the walls were treated with Dirichlet boundary condition for the velocity field (no slip $u=0$) and the Neumann condition ($\partial p/\partial n=0$) for the pressure field. Simulations were carried out with a time step $dt=0.15$ using the implicit Crank-Nicolson numerical scheme for discretizing time derivatives and second order Gaussian discretization for space derivatives.

## Supplementary Information

Supplementary Information 1.

Supplementary Information 2.

Supplementary Information 3.

Supplementary Information 4.

Supplementary Information 5.

Supplementary Information 6.

## Data Availability

The datasets used and/or analysed during the current study available from the corresponding author on reasonable request.
