# Supplementary material for: Wave dispersion in moderate channel turbulence
Source: Sci Rep. 2023 Apr 26;13:6801. doi: 10.1038/s41598-023-32978-7 (PMC10133326; doi:10.1038/s41598-023-32978-7)
Supplement: Supplementary file 6 — Supplementary Information 6. [file 41598_2023_32978_MOESM6_ESM.pdf]

Ocean-like dispersion in moderate channel turbulence

Chiara Pilloton[1] , Claudio Lugni[1,4,5], Giorgio Graziani[3], and Francesco Fedele[2*]

[1]CNR-INM, Institute of Marine Engineering, Via di Vallerano 139, 00128 Roma, Italy
[2]School of Civil and Environmental Engineering, Georgia Institute of Technology, Atlanta, USA
[3]Department of Mechanical and Aerospace Engineering, Sapienza Universitá di Roma, Roma, Italy
[4]NTNU-AMOS, Center for Autonomous Marine Operation Systems, Trondheim, Norway
[5]Institute of Marine Hydrodynamics, Harbin Engineering University, Harbin, China
[*]fedele@gatech.edu

Supplementary Video Legend

Movie S1 Space-time evolution of the mean spanwise vorticity field $\overline{\varpi}_z(x,y,t)$.

Movie S2 Space-time evolution of a 2D QD vortical packet of spanwise vorticity that focuses near the wall.

Movie S3 Space-time evolution of a 2D QD vortical packet of spanwise vorticity that focuses near the channel's centre.

Movie S4 Space-time evolution of a 2D QD vortical packet of streamwise vorticity that focuses near the wall.

Movie S5 Space-time evolution of a 2D QD vortical packet of streamwise vorticity that focuses near the channel's centre.
